# Is hereditary angioedema associated with deficits in emotion regulation? A quantitative study in adult patients

**DOI:** 10.1186/s13023-026-04198-5

**Published:** 2026-02-12

**Authors:** Christelle Duprez, Véronique Christophe, Isabelle Citerne, Michel Raguet, Louise Richez, Sébastien Sanges, David Launay

**Affiliations:** 1 Univ. Lille, CNRS, UMR 9193 - SCALab - Sciences Cognitives et Sciences Affectives, Lille, F-59000 France; 2 Université Claude Bernard Lyon 1, Centre de Recherche en Cancérologie de Lyon - UMR Inserm 1052 - CNRS 5286 - UCBL - CLB, Lyon, France; 3 Département de Sciences Humaines et Sociales, Centre Léon Bérard, Lyon, France; 4 CHU Lille, Département de Médecine Interne et Immunologie Clinique, Lille, F-59000 France; 5 Centre de Référence des Angiœdèmes à Kinines (CREAK), Lille, F-59000 France; 6 AMSAO (association des malades souffrant d’angioedèmes), Paris, France; 7 Univ. Lille, U1286 - INFINITE Institute for Translational Research in Inflammation, Lille, F-59000 France; 8 Inserm, Lille, F-59000 France

**Keywords:** Hereditary angioedema, Emotion regulation, Alexithymia, Quality of life, Anxiety, Depression, Parental bonding

## Abstract

**Background:**

Patients with hereditary angioedema (HAE) exhibit emotional distress and a deteriorated quality of life. Emotional distress is associated with impaired emotional regulation, including alexithymia, which is characterized by difficulties in recognizing and expressing emotions. Our study assessed the presence of alexithymia in patients with HAE to determine whether patients with this disease experience difficulties and specific learning in emotion regulation. Thirty-nine adult patients with HAE answered a self-report questionnaire measuring: 1) alexithymia (*Toronto Alexithymia Scale*-20), 2) anxiety and depression symptoms (*Hospital Anxiety and Depression Scale*), 3) quality of life (*Angioedema Quality of Life Questionnaire*), 4) emotion regulation skills (*Emotion Regulation Skills Questionnaire*), 5) difficulties in emotion regulation (*Difficulties in Emotion Regulation Scale*), and 6) perceived quality of parental care (*Parental Bonding Instrument*).

**Results:**

No alexithymia was detected in 39.5% of patients, 36.8% of patients had definite alexithymia and 23.7% had possible alexithymia. Alexithymia was associated with fewer skills overall and more difficulties with emotion regulation, as well as with more depressive symptoms and lower quality of life in terms of fatigue/mood. Emotion regulation skills and difficulties also appeared to be associated with overprotection in terms of perceived paternal style.

**Conclusions:**

This study shows a low prevalence of definite alexithymia in HAE patients and non-deficient emotion regulation. The association between alexithymia and depressive symptomatology suggests the potential role of interventions targeting emotion regulation features

## Background

Hereditary angioedema (HAE) is a rare genetic disorder resulting from excessive bradykinin production linked to a genetic mutation. It affects 1 in 50,000 people, men and women equally, and can occur at any age. HAE is often associated with C1-inhibitor (C1INH) deficiency due to mutations in the *SERPING1* gene (HAE-C1INH), that can modify both the serum levels and function of the protein (HAE-C1INH type 1) or only its protease activity (HAE-C1INH type 2). More rarely, HAE is associated with other genetic mutations (e.g. *FXII*, *PLG*, *KNG*) that do not impact C1INH levels and function (HAE-nC1INH). In all cases, the disease progresses in acute attacks characterized by localized swelling, known as angioedema. These attacks can affect the face, lower and upper limb extremities, digestive tract or respiratory airways. In case of a laryngeal attack, death by asphyxia can occur in up to 25% of cases in the absence of specific treatment. Medical management consists of a combination of on-demand treatment to treat attacks, long-term prophylaxis to prevent them and short-term prophylaxis administered right before high-risk procedures (such as surgeries, dental procedures, and endoscopies) [1, 2]. Depending on attack frequency, severity and patient preference, treatment strategy will consist of either a long-term prophylaxis associated with on-demand treatment, or on-demand treatment only. Disease activity and severity differ from patient to patient and, over time, may both evolve in the same patient [1, 2]. HAE attacks may be spontaneous, or triggered by factors such as infections, medications, hormonal factors, physical trauma, or medical procedures (e.g., dental surgery). Emotions (e.g., fear, anxiety, stress) are also among the triggers that patients reported the most [3–8].

Psychological factors play a complex role in HAE: emotions are reported as triggers for attacks, while the attacks themselves have an emotional impact [9]. Of patients with HAE, 24%–39% present with mild to severe depression, and 15%–49% with anxiety [9–13]. The majority of HAE patients, up to 64%, also have higher levels of perceived stress than the general population [9]. Depending on disease activity, HAE can also impact professional, educational, and social activities [9, 13–19]. However, in recent years, treatments for HAE have evolved considerably with the arise of kallikrein inhibitors (lanadelumab, berotralstat), and diagnosis delays are becoming shorter thanks to increased disease awareness [20, 21]. By drastically reducing or even completely eliminating attacks, kallikrein inhibitors help to limit HAE’s impact on daily life, improving quality of life [9, 22]. As a result of medical management, 99% of patients report a reduction in the impact of the disease on their daily lives since their diagnosis, and for 32%, the disease no longer has any impact at all [20].

Emotional distress, such as anxiety and depression, is associated with difficulties in emotion regulation i.e., the ability to automatically or voluntarily influence our emotions, whether positive or negative [23–27]. Emotion regulation can also be conceptualized as the implementation of a set of skills acquired during social learning, such as the competence to become aware of one’s emotions, identify them, and name them correctly [28]. Effective emotion regulation involves flexibility in the use of different emotion regulation strategies [29, 30], as well as the awareness, understanding, acceptance of emotions, the ability to continue to act according to previously set goals and control impulsive behaviors under the influence of emotion. The absence of any of these abilities indicates the presence of emotion regulation difficulties [24]. Alexithymia, which is characterized by difficulties in identifying and describing one’s feelings, difficulties in distinguishing them from the bodily sensations of emotional activation, and by a tendency to focus one’s attention on external events [31–33], for a review, see can be seen as a deficit in emotion regulation [32]. Alexithymia and emotional distress are linked via emotion regulation strategies [34–36], and alexithymia is associated with less flexible and therefore less adaptive emotion regulation, i.e., emotional dysregulation [35, 37].

Emotion regulation is learned during child development [38, 39]. An affectionate, available, and empathetic parenting style seems to be associated with good emotion regulation abilities in children, whereas a parenting style characterized by controlling, intrusive, and overprotective behavior when facing emotions is associated with emotion regulation difficulties in emotion regulation [38–40]. Furthermore, perceived parenting styles during childhood or adolescence are associated with specific mental health issues in adulthood (for example, depression [41]); and with emotion regulation difficulties more generally [42]. In particular, parenting styles are associated with alexithymia, especially perceived maternal style is most associated with alexithymia, with perceived paternal style having a minimal impact on alexithymia [43].

An Italian study has highlighted that children with HAE report a greater number of stressful events in the previous year compared with children of the same age who do not have HAE and had above-average alexithymia scores [5]. While the fact that children with HAE report more stress may be linked to lower emotion regulation capacities, as suggested by Freda et al. [5], it may also imply that children with HAE find stress difficult to recognize and manage effectively. On the other hand, the belief that stress can trigger disease attacks may lead the parents and physicians of children with HAE to suggest them, implicitly or explicitly, that they should avoid stressful or intense emotional events. This could result in children inhibiting their ability to be aware of and express their emotions, consequently diminishing their ability to cope with emotional events [5]. Ultimately, and paradoxically, this would lead to greater vulnerability to stress. In line with Freda et al. [5], Savarese et al. [44] showed that among 28 children with HAE aged 6 to 17 years old, 72% had a significant alexithymia score and perceived stress was positively correlated with the alexithymia score.

Furthermore, alexithymia is only partially observed in adult HAE patients. In 28 adult patients Savarese et al. [45] assessed the level of alexithymia with the TAS-20, a self-report questionnaire measuring alexithymic tendencies and two emotion regulation strategies (positive reappraisal and expressive suppression). Their data showed an absence of alexithymia in 78% of patients surveyed, definite alexithymia in only 11% of patients, and possible alexithymia in a further 11%. In terms of emotion regulation, HAE patients did not differ from those of the general population. Savarese et al. [45] concluded that alexithymia and emotion regulation deficits characterize the experiences of children with chronic illnesses due to their developmental stage, whereas adults are able to identify and regulate their emotions.

**Fig. 1:**
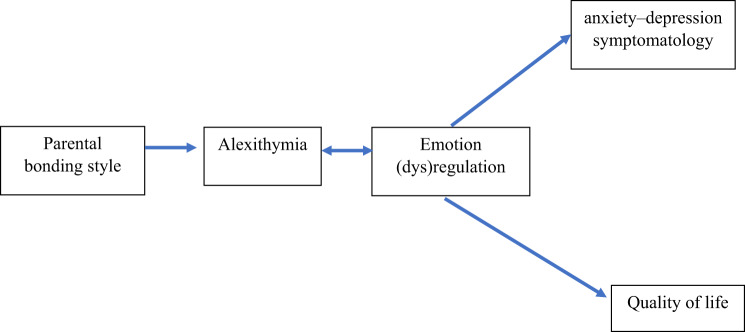
Theoretical model

The current study aimed to complement this finding with French adult patients with HAE by focusing on emotional processes through the assessment of emotion regulation skills and difficulties. Indeed, it is possible that adult patients show difficulties in emotion regulation (e.g., difficulties in identifying their emotional states) and that patients with higher levels of alexithymia have fewer emotion regulation skills and more specific emotion regulation difficulties than patients with lower level of alexithymia. The objective was therefore to study the frequency of emotion regulation deficits in the form of alexithymia, as well as the association between alexithymia, emotion regulation skills, and emotion regulation difficulties, in order to identify whether any profiles emerge in French adult patients with HAE. Based on literature (Fig. 1), we hypothesize that parenting style that does not promote emotional experience (i.e., overprotection) will be associated with more alexithymia, which will in turn be associated with more difficulties and less skills in emotion regulation, higher emotional distress and lower quality of life. The impact of kallikrein inhibitors on alexithymia, emotional distress, emotion regulation and quality of life was also investigated as secondary objective.

## Methods

### Study design

This single-center, observational study was offered to adult patients with biologically proven HAE-C1INH-Type 1 or -Type 2 who were aged 18+ years and had a medical follow-up appointment already scheduled in the relevant hospital department during the study period. Inclusion took place from November 2021 to November 2023 in the Internal Medicine and Clinical Immunology Department of Lille University Hospital, a national reference center for bradykinin-mediated angioedema. The study obtained approval from the University of Lille Ethics and Research Committee in November 2019 (reference 2019–393-S78). The data were collected in accordance with the European Union General Data Protection Regulation (GDPR). The study has been declared in the Data Protection Officer’ register for the University of Lille (reference 2019–085).

### Procedure

The questionnaires were administered in the Department during a scheduled medical follow-up appointment. Three weeks before their appointment, patients who met the inclusion criteria were contacted by telephone and introduced to the study. Interested patients were sent an information letter and consent form in advance, allowing them sufficient time to read the documents and reflect before their appointment. Patients who agreed in principle were asked to arrive early for their medical follow-up consultation to participate in the study.

On the day of the appointment, the investigator (a research assistant in psychology) ensured that the patient had read and understood the information letter, and collected the patient’s written consent. The patient was then asked to complete a pencil-and-paper self-administered questionnaire, as well as a sociodemographic data sheet collecting age, gender, and socioeconomic and educational data. The patient was also asked to fill in a medical data sheet, providing the number of HAE attacks, their location, and the various treatments already received and in progress. The questionnaire took around an hour to complete. Once the questionnaire was completed, the patient was debriefed and allowed to ask any questions they had before going to their medical consultation. The physician was not present when the questionnaire was administered, and the confidentiality of the patient’s answers was guaranteed.

### Measures

Patients were asked to complete the following self-report questionnaires:

*Toronto Alexithymia Scale* (TAS-20 [46, 47], a self-report questionnaire measuring alexithymic tendencies. The scale comprises 20 items, assessing three dimensions*: Difficulties in verbalizing emotional states to others* (TAS FACTOR 1, five items, e.g., “I have difficulty finding words that correspond well to my feelings”), *Difficulties in identifying feelings* (TAS FACTOR 2, seven items, e.g., “I often can’t see my feelings clearly”), and the *Tendency to turn attention to external elements* (TAS FACTOR 3, operative thinking, eight items, e.g., “I prefer to let things happen rather than understand why they’ve turned out the way they have”). The responses are based on a 5-point Likert scale, scored from 1 = “complete disagreement” to 5 = “complete agreement.” Five items are reverse-scored (Items 4, 5, 10, 18, 19). The total alexithymia score is the sum of all items, with a possible range from 20 to 100. A score can also be calculated for each of the three dimensions. A higher score indicates a higher level of alexithymia. An overall score greater than or equal to 61 indicates the presence of alexithymia; a score between 52 and 60 indicates possible alexithymia, and a score less than or equal to 51 indicates the absence of alexithymia. As the TAS-20 is not a diagnostic tool, scores should be interpreted as measuring alexithymic tendencies rather than identifying clinical cases.

*Hospital Anxiety and Depression Scale* (HADS [48]). This scale, which is commonly used in clinical populations, screens for anxiety and depressive symptoms. It comprises 14 items, rated from 0 to 3. Seven questions relate to anxiety (Total A) and seven to depression (Total D), giving two scores (the maximum score for each is 21). Patients respond according to how they felt over the previous week. The higher the score, the more likely symptomatology is present (anxiety symptomatology, depressive symptomatology). A score less than or equal to seven indicates no symptomatology, a score between 8 and 10 indicates the possible presence of symptomatology, a score between 11 and 14 indicates moderate symptomatology, and a score between 15 and 21 indicates severe symptomatology [49].

*Angioedema Quality of Life Questionnaire* (AE-QoL [19], which measures quality of life using 17 items covering four quality of life domains: *Physical functioning, Fatigue/mood, Fears/shame, and Diet*. Patients are asked to indicate on a 5-point scale (from 1 = “never” to 5 = “very often”) the extent to which the various proposals applied to their experience over the past four weeks, whether or not they had an angioedema attack during this period. The scores for the subdimensions, as well as the overall score, are the linear transformation, from 0 to 100, of the raw scores. The higher the score, the worse the quality of life.

*Emotion Regulation Skills Questionnaire* (ERSQ [50]). This tool measures emotion regulation skills. Respondents indicate the extent to which they have applied emotion regulation skills/managed their emotions over the previous week. The scale comprises 27 items, divided into nine subscales, with three items per skill type: *Awareness of one’s emotions* (AWARENESS, e.g., “I was able to consciously pay attention to my emotions”), *Sensations* (SENSATIONS, e.g., “My physical sensations were a good indicator of how I was feeling”), *Clarity* (CLARITY, e.g., “I was able to identify my emotions”), *Understanding* (UNDERSTANDING, e.g., “I understood my emotional reactions”), *Modification* (MODIFICATION, e.g., “I was able to consciously provoke positive emotions”), *Acceptance* (ACCEPTANCE, e.g., “I was able to accept my negative emotions”), *Tolerance* (TOLERANCE, e.g., “I was able to overcome my negative emotions”), *Ability to confront painful situations if necessary to achieve a goal* (CONFRONTATION, e.g., “I did what I wanted to do, even if I had to face negative emotions along the way”), and *Self-support* (SELF-SUPPORT, e.g., “I tried to reassure myself during trying situations”). Each question is answered using a 5-point Likert scale, ranging from 0 = “not at all” to 4 = “almost always.” The higher the subscale score, the more the patient implements the skill being assessed. An overall score can be calculated by averaging the sum of all the items [50]. The higher the score, the more efficient the emotion regulation.

*Difficulties in Emotion Regulation Scale—16-item version* (DERS-16 [51]), which measures difficulties in emotion regulation. Patients completed the questionnaire by indicating, using a Likert scale ranging from 1 (“almost never”) to 5 (“almost always”), the extent to which the different statements applied to them. The 16-item scale measures *Difficulties in accepting negative emotions* (NON-ACCEPTANCE, three items, e.g., “When I am upset, I feel ashamed of feeling such an emotion”), *Continuing to act according to the goals that one had previously set* (GOALS, three items, e.g., “When I am upset, I have difficulty finishing a task”), *Controlling impulsive behaviors under the influence of emotion* (IMPULSE, three items, e.g., “When I am upset, I become uncontrollable”), *Accessing regulation strategies perceived as effective* (STRATEGIES, five items, e.g., “When I am upset, I think that I will stay like this for a very long time”), and *Being aware of one’s emotions* (AWARENESS, two items, e.g., “I have difficulty making sense out of my feelings”). The overall score is the sum of the items and ranges from 16 to 80. The higher the score, the more difficulties in emotion regulation are present.

*Parental Bonding Instrument* (PBI [52, 53]), which measures perceived parenting style during childhood and adolescence. The instrument comprises two parts, one concerning perceptions of the mother’s parenting style and the other concerning perceptions of the father’s parenting style. Each part is composed of 25 statements concerning various parental behaviors and attitudes, with the only difference being whether the instruction concerns the mother or the father. Perceived parental bonding is measured on two dimensions: CARE (warmth of the relationship, perceived emotional involvement, e.g., “my father/mother seemed to understand my problems and worries”), and OVERPROTECTION (parental control, e.g., “my father/mother overprotected me”). In this study, patients were asked to respond by referring to their memories of their mother/father, accumulated during the first years of their life, childhood, and adolescence. Using a Likert-type scale, they had to indicate to what extent their mother/father’s behavior or attitude was more or less similar to what is described in the statement. The questions are separate for the mother and the father, with the responses to each of the 25 statements coded from 0 to 3. There are two scores regarding the maternal style (maternal care, maternal overprotection), and two scores regarding the paternal style (paternal care, paternal overprotection). Based on the scores for the two dimensions, four types of parental bonding can be distinguished: *affectionate constraint* (high care and high protection), *affectionless control* (high protection and low care), *optimal parenting* (high care and low protection) and *neglectful parenting* (low care and low protection). For mothers, a care score of 27 and a protection score of 13.5 reflect high and low scores, respectively. For fathers, a care score of 24 and a protection score of 12.5 reflect high and low scores, respectively.

### Statistical analysis

Means and standard deviations were calculated for the continuous variables, and the categorical variables were described by percentages. The normality of the distributions was tested using the Shapiro–Wilk test at the threshold of alpha = 0.10 and verified graphically by histograms, with an examination of the asymmetry and kurtosis coefficients (between −1 and +1). Depending on the normality of the distributions, parametric (Pearson correlations) or non-parametric (Spearman correlation) inferential analyses were conducted. Correlations between the patient’s current age, duration of illness, gender (recoded: 1 for women, 2 for men), and the overall TAS-20 score—the variable of interest in the study—were also calculated to identify possible covariates to be taken into account (partial correlations).

The links between difficulties in emotion regulation (DERS-16) and alexithymia (TAS-20), and between emotion regulation skills (ERSQ) and alexithymia (TAS-20), were studied using correlations, across the whole sample. Based on the existing literature, the correlations between difficulties in emotion regulation (DERS-16) and anxiety–depression symptomatology (HADS) on the one hand, and difficulties in emotion regulation (DERS-16) and quality of life (AE-QoL) on the other hand, were calculated, as were the links between emotion regulation skills (ERSQ) and anxiety–depression symptomatology (HADS), and between emotion regulation skills (ERSQ) and quality of life (AE-QoL). As parenting styles have been documented in the literature to influence emotion regulation abilities (e.g., Morris et al., 2007, 2017), correlations between the PBI, DERS-16, and ERSQ scores were also calculated. The correlations were interpreted as follows: 0–0.19: very weak, 0.2–0.39: weak, 0.40–0.59: moderate, 0.6–0.79: strong, > 0.80: very strong.

Alexithymia (TAS-20), anxiety–depression symptomatology (HADS), difficulties in emotion regulation (DERS-16), emotion regulation skills (ERSQ) and quality of life (AE-QoL) were compared between patients under kallikrein inhibitors (*n* = 15) versus patients with other treatments (*n* = 24). Kolmogorov-Smirnov test was conducted in place of U Mann-Whitney test because the sample size was less than 25 per group [54].

All analyses were conducted using IBM SPSS Statistics, version 29 software, at the alpha threshold = 0.05.

## Results

### Patients

Thirty-nine patients (59% female) with a mean age of 49.92 years (*SD* = 14.97, range = 19–76 years) participated in the study. The patients’ sociodemographic and medical data are presented in Table 1. The majority of patients had HAE-C1INH-Type1 (97.40%), and the mean age at diagnosis was 22.87 years (*SD* = 11.99, range = 6–55 years). On average, patients reported the occurrence of three attacks in the last six months (*SD* = 4.76, range = 0–20), and 30.80% the occurrence of a severe attack in the last six months. For the majority of patients (87.20%), the sites involved were the abdomen and the upper and lower limbs. Stress was reported as a trigger for attacks by 84.21% of patients, before physical trauma (55.26%). Regarding their medical treatment, 38.50% of patients were receiving kallikrein inhibitor treatment at the time of the study (13 patients receiving lanadelumab, 33.33% and 2 patients were on berotralstat, 5.12%), for an average of 1.5 years (*M* = 544.27 days, *SD* = 348.94, min = 23 days, max = 1444 days).

**Table 1:** Demographics and clinical data of patients

**Age** *(M, SD, range)*	49.92 (14.97) Range: 19 - 76
**Gender** (n, %)	
Males	16 (41%)
Females	23 (59%)
**Professional activity** (*n*, %)	
Craftsmen, shopkeepers and company directors	1 (2.60%)
Managers and higher intellectual professions	6 (15.40%)
Intermediate professions	3 (7.70%)
Employees	7 (17.90%)
Manual workers	3 (7.70%)
Non-active	9 (23.10%)
Students	1 (2.60%)
Retired	9 (23.10%)
**Education** (*n*, %)^a^	
No diploma	3 (8.33%)
Junior school certificate	2 (5.55%)
Vocational diploma	11 (30.55%)
High school diploma	11 (30.55%)
Bachelor degree	3 (8.33%)
Master degree	6 (16.67%)
**Marital status** (*n*, %)	
Single	8 (20.51%)
In a couple	31 (79.5%)
**Age at diagnosis, in years** (*M, SD, range)*^*b*^	22.87 (11.99) Range: 6 - 55
**Type of disease** (*n*, %)	
Type 1	38 (97.40%)
Type 2	1 (2.60%)
**Length of the disease, in years** (*M, SD, range*)^c^	27.53 (17.04) Range: 0 - 62
**Biology at diagnosis** *(n, %)*	
C1 inhibitor (mg/L) (*M, SD*)	78,74 (46.26%)
C4 assay (mg/L) (*M, SD*)	70,95 (63.77%)
**Location of angioedema already presented by the patient** (*n*, %)	
Otorhinolaryngeal with laryngeal involvement	16 (41.02%)
Otorhinolaryngeal without laryngeal involvement	8 (20.51%)
Face	25 (64.10%)
Abdomen	34 (87.20%)
Upper limbs	34 (87.20%)
Lower limbs	34 (87.20%)
External genitals	18 (47.37%)
Other (digestive tract, bladder, rectum, back)	3 (7.89%)
Number of attacks over the last 6 months (*M, SD range*)^d^	3.23 (4.76) Range: 0 - 20
Presence of a severe attack in the last 6 months (*n*, %)^e^	12 (30.8%)
**Attacks triggers** (*n*, %)^f^	
Stress	32 (84.21%)
Physical trauma	21 (55.26%)
Physical activity	18 (47.37%)
Drugs	9 (23.68%)
Surgery	13 (34.21%)
Environmental factors	11 (28.95%)
Infection	13 (34.21%)
Others	10 (26.31%)
**Attacks treatments– treatments already received** (*n*, %)^g^	
Icatibant	25 (69.44%)
Tranexamic acid	1 (2.70%)
C1 inhibitor concentrate	6 (16.21%)
Therapeutical abstention	7 (18.91%)
**Long-term treatments – treatments already received** (*n*, %)^h^	
Danazol	15 (39.47%)
Tranexamic acid	4 (10.52%)
Progestogen	2 (5.26%)
C1 inhibitor concentrate	6 (15.79%)
Lanadelumab	10 (26.31%)
Berotralstat	9 (23.68%)
**Intake of kallikrein inhibitor at the time of the inclusion in the study** *(n, %)*	15 (38.50%)
**Lenght of the treatment with kallikrein inhibitor, in days** *(M, SD, range)*	544.27 (348.94) Range: 23 - 1444
**participation in therapeutic patient education sessions** *(n, %)*	19 (48.71%)
**Psychological support** *(n, %)*	2 (5.13%)

**Abbreviations**: *N:* number of subjects; *M:* mean; *SD:* standard deviation. **Notes.**^a^ the diploma level was not completed by 3 patients; ^b^ age at diagnosis was not completed for 1 patient; ^c^consequently, the duration of illness was not calculable for 1 patient; ^d^ Number of attacks over the last 6 months: 9 missing values; ^e^ Presence of a severe attack/relapse in the last 6 months: 1 missing value; ^f^Attacks triggers: a patient did not complete this information. Among « other triggers », patients reported: fatigue (*n* = 3), ovulation (*n* = 1), death (*n* = 1); food (*n* = 2), vaccine (*n* = 1) or could not identify triggers (*n* = 2); ^g^ Attacks treatments: 3 missing values concerning icatibant, 2 missing values concerning the other responses; ^h^ Long-term treatments: this information was not available for one patient

Table 2 presents the scores on the different scales as well as score interpretations based on the cut-off. Regarding the TAS-20 score (*M* = 54.95, *SD* = 14.56), 39.5% of patients had no alexithymia (TAS-20 score under or equal to 51), 36.8% had definite alexithymia (TAS-20 score over or equal to 61) and 23.7% had possible alexithymia (TAS-20 score between 52 and 60). The majority of patients did not have anxiety (53.8%) or depression (76.9%). The mean score was *M* = 8.77 (*SD* = 4.72) on the HADS A (HADS anxiety scale) and *M* = 4.62 (*SD* = 3.78) on the HADS D (HADS depression scale). Based on the cut off, most patients (53.8%) show no anxiety, and no depression (76.9%). The overall quality of life score was 39.48 (*SD* = 21.94), indicating a fairly good quality of life.

**Table 2:** Descriptive data

Scale	n (%)
**TAS 20** ^**a**^	
Category based on global score *(n, %):*	
Absence of alexithymia (score TAS ≤ 51)	15 (39.50%)
Possible alexithymia (52 < score TAS < 60)	9 (23.70%)
Presence of alexithymia (score TAS ≥ 61)	14 (36.80%)
Factor 1 *(M, SD, range)*	18.55 (7.82) Range: 7 - 35
Factor 2 *(M, SD, range)*	15.42 (4.05) Range: 5 - 22
Factor 3 *(M, SD, range)*	20.97 (5) Range: 9 - 29
Global score *(M, SD, range)*	54.95 (14.56) Range: 22–85
**HADS**	
Category based on the HADS A score *(n, %)*	
Absence of anxiety (score < 7)	21 (53.80%)
Possible presence (8 < score < 10)	6 (15.40%)
Moderate anxiety (11 < score < 14)	5 (12.80%)
High anxiety (15 < score < 21)	7 (17.90%)
Category based on the HADS D score *(n, %)*	
Absence of depression (score < 7)	30 (76.90%)
Possible presence (8 < score < 10)	6 (15.40%)
Moderate depression (11 < score < 14)	2 (5.10%)
High depression (15 < score < 21)	1 (2.60%)
**AEQOL** ^**b**^ *(M, SD, range)*	
Overall score	39.48 (21.94) Range: 5.88–82.35
functioning	27.14 (27.99) Range: 0 - 87.5
diet	34.12 (29.41) Range: 0 - 87.5
fatigue/mood	44.59 (27.59) Range: 0 - 100
fears/shame	45.25 (28.65) Range: 0 - 95.83
**ERSQ** ^**c**^ *(M, SD, range)*	
overall mean	2.5 (0.63) Range: 0.78 - 3.44
awareness	2.54 (0.82) Range: 0.33 - 4
sensations	2.69 (0.71) Range: 0.67 - 4
clarity	2.61 (0.74) Range: 0.67 - 3.67
understanding	2.47 (0.91) Range: 0.33–4
acceptance	2.21 (0.74) Range: 0.67–3.33
tolerance	2.31 (0.84) Range: 0.33–4
self support	2.68 (0.78) Range: 0–3.67
confrontation	2.66 (0.62) Range: 1–4
modification	2.31 (0.94) Range: 0–3.67
**DERS** ^**d**^*(M, SD, range)*	
overall score	32.89 (14.41) Range: 19 - 79
clarity	4.39 (2.24) Range: 1 - 10
goals	7.42 (3.05) Range: 3 - 15
impulse	5.18 (3.13) Range: 3–15
strategies	9.76 (4.97) Range: 5–25
non acceptance	6.13 (3.37) Range: 3–15
**PBI** ^**e**^ *(M, SD, range)*	
Care - mother	26.59 (6.58) Range: 12–36
Overprotection - mother	11.89 (6.68) Range: 2–27
Care - father	23.08 (10.08) Range: 0–36
Overprotection - father	11.68 (6.47) Range: 0–28

**Notes.** ^a^ *N* = 38, ^b^ *N* = 37, ^c^ *N* = 37, ^d^ *N* = 38, ^e^ *N* = 37. **Abbreviations**: TAS 20: Toronto Alexithymia Scale-20, HADS: Hospital Anxiety and Depression Scale, AEQoL: Angioedema Quality of Life Questionnaire; ERSQ: Emotion Regulation Skills Questionnaire; DERS: Difficulties in Emotion Regulation Scale; PBI: Parental Bonding Instrument; N: number of subjects; M: mean; SD: standard deviation

Normality was rejected for all the variables except ERSQ tolerance, ERSQ confrontation, TAS factor 1, TAS global factor, AE-QoL fatigue, AE-QoL global, and PBI paternal overprotection. The asymmetry and kurtosis coefficients were also poor for the variables ERSQ self-support, DERS impulse, DERS strategies, DERS global, PBI maternal care, PBI maternal overprotection, PBI paternal overprotection, and PBI paternal care. These results, coupled with the fact that the sample was small, led us to conduct non-parametric analyses on all the variables (Spearman correlations). These analyses also showed that age (rho = 0.02, *p* = 0.90), gender (rho = −0.03, *p* = 0.87), and disease duration (rho = 0.06, *p* = 0.73) were not correlated with the overall TAS-20 score, so the correlations were calculated without controlling for these variables.

## Correlations

### Alexithymia and emotion regulation

The data presented in Table 3 show a negative and significant albeit moderate association between the global alexithymia score and emotion regulation skills in terms of global ERSQ score (rho = −0.56, *p* < 0.01), awareness (rho = −0.56, *p* < 0.01), clarity (rho = −0.50, *p* < 0.01), acceptance (rho = −0.39, *p* < 0.05), tolerance (rho = −0.52, *p* < 0.01), self-support (rho = −0.41, *p* < 0.05), confrontation (rho = −0.37, *p* < 0.05), and modification (rho = −0.45, *p* < 0.01). The negative association between the TAS global score and ERSQ understanding was strong (rho = −0.62, *p* < 0.01). The link between TAS-20 and the sensations dimension was non-significant (Table 3).

**Table 3:** Correlations between ERSQ, DERS and TAS scores

	1.	2.	3.	4.	5.	6.	7.	8.	9.	10.	11.	12.	13.	14.	15.	16.	17.	18.	19.	20.
1. ERSQ global score	–																			
2. ERSQ awareness	0.80**	–																		
3. ERSQ sensations	0.60**	0.43**	–																	
4. ERSQ clarity	0.79**	0.69**	0.44**	–																
5. ERSQ understanding	0.84**	0.67**	0.47**	0.79**	–															
6. ERSQ acceptance	0.70**	0.55**	0.29	0.42*	0.48**	–														
7. ERSQ tolerance	0.80**	0.68**	0.42**	0.56**	0.58**	0.73**	–													
8. ERSQ self support	0.73**	0.59**	0.43**	0.65**	0.69**	0.38*	0.37*	–												
9. ERSQ confrontation	0.71**	0.46**	0.23	0.51**	0.49**	0.52**	0.61**	0.56**	–											
10. ERSQ modification	0.78**	0.53**	0.51**	0.53**	0.73**	0.40*	0.54**	0.68**	0.49**	–										
11. DERS global	−0.37*	−0.30	0.07	−0.15	−0.28	−0.33*	−0.45**	−0.25	−0.32	−0.43**	–									
12. DERS clarity	−0.38*	−0.30	−0.04	−0.30	−0.42**	−0.41*	−0.49**	−0.20	−0.21	−0.30	0.57**	–								
13. DERS goals	−0.11	−0.06	0.23	0.01	−0.10	−0.11	−0.18	−0.06	−0.13	−0.26	0.86**	0.34*	–							
14. DERS impulse	−0.45**	−0.46**	−0.01	−0.31	−0.46**	−0.34*	−0.45**	−0.32	−0.30	−0.36*	0.78**	0.45**	0.69**	–						
15. DERS strategies	−0.41*	−0.34*	−0.01	−0.16	−0.29	−0.31	−0.40*	−0.36*	−0.38*	−0.52**	0.88**	0.33*	0.78**	0.71**	–					
16. DERS non acceptance	−0.24	−0.26	0.02	−0.11	−0.16	−0.08	−0.30	−0.16	−0.08	−0.22	0.67**	0.34*	0.50**	0.53**	0.51**	–				
17. TAS global score	−0.56**	−0.56**	−0.21	−0.50**	−0.62**	−0.39*	−0.52**	−0.41*	−0.37*	−0.45**	0.41*	0.63**	0.2	0.58**	0.28	0.32	–			
18. TAS factor 1	−0.49**	−0.52**	−0.11	−0.42**	−0.54**	−0.23	−0.49**	−0.34*	−0.35*	−0.43**	0.48**	0.62**	0.30	0.64**	0.32*	0.37*	0.95**	–		
19. TAS factor 2	−0.37*	−0.38*	−0.1	−00.25	−0.43**	−0.42**	−0.38*	−0.31	−0.15	−0.38*	0.39*	0.62**	0.13	0.46**	0.24	0.34*	0.87**	0.81**	–	
20. TAS factor 3	−0.50**	−0.49**	−0.33*	−0.56**	−0.56**	−0.28	−0.36*	−0.44**	−0.36*	−0.34*	0.08	0.33*	0.002	0.22	0.09	−0.01	0.69**	0.52**	0.40*	–

Note. * *p* < 0.05, ** *p* < 0.01. Abbreviations: TAS 20: Toronto Alexithymia Scale-20, ERSQ: Emotion Regulation Skills Questionnaire; DERS: Difficulties in Emotion Regulation Scale

Concerning emotion regulation difficulties, the data showed a positive and significant albeit moderate association between the overall DERS-16 score and the overall TAS-20 score (rho = 0.41, *p* < 0.05), and TAS-20 and impulse (rho = 0.58, *p* < 0.01). The association was positive and strong between the overall TAS-20 score and the clarity dimension (rho = 0.63, *p* < 0.01). The links between alexithymia and goals, strategies, and non-acceptance were non-significant (Table 3).

### Alexithymia and anxiety–depression symptomatology

The links between the TAS-20 and HADS scores are shown in Table 4. Alexithymia (overall TAS-20 score) was positively and moderately correlated with depressive symptomatology (rho = 0.45, *p* < 0.01) but not with anxiety symptomatology (rho = 0.26, ns).

**Table 4:** Correlations between TAS, HADS and AEQoL scores

	1.	2.	3.	4.	5.	6.	7.	8.	9.	10.	11.
1. TAS global score	–										
2. TAS factor 1	0.95**	–									
3. TAS factor 2	0.87**	0.81**	–								
4. TAS factor 3	0.69**	0.52**	0.40*	–							
5. HADS anxiety	0.26	0.24	0.28	0.04	–						
6. HADS depression	0.45**	0.47**	0.38*	0.25	0.42**	–					
7. AE-QoL overall score	0.27	0.25	0.29	0.04	0.64**	0.42*	–				
8. AE-QoL fears/shame	0.16	0.12	0.28	−0.05	0.54**	0.24	0.80**	–			
9. AE-QoL functionning	0.12	0.09	0.12	0.08	0.49**	0.26	0.68**	0.36*	–		
10. AE-QoL diet	0.09	0.08	0.14	0.08	0.41*	0.28	0.66**	0.24	0.59**	–	
11. AE-QoL fatigue/mood	0.45**	0.45**	0.36*	0.21	0.48**	0.44**	0.80**	0.51**	0.33*	0.54**	–

Note. * *p* < 0.05, ** *p* < 0.01. Abbreviations: TAS 20: Toronto Alexithymia Scale-20, HADS: Hospital Anxiety and Depression Scale, AEQoL: Angioedema Quality of Life Questionnaire

### Alexithymia and quality of life

In terms of quality of life, only the fatigue/mood dimension of the AE-QoL was associated, positively and moderately, with the TAS-20 score (rho = 0.45, *p* < 0.01).

### Emotion regulation and anxiety–depression symptomatology

As shown in Table 5, only emotion regulation skills (ERSQ) in terms of acceptance (rho = −0.35, *p* < 0.05) and tolerance (rho = −0.45, *p* < 0.01) were negatively associated with anxiety symptoms (HADS A score). The overall ERSQ score and all the ERSQ dimensions except for the sensations dimension were negatively associated with depressive symptoms (HADS D score), with the association between the overall ERSQ (rho = −0.57, *p* < 0.01), the ERSQ confrontation (rho = −0.58, *p* < 0.01) and the self-support (rho = −0.56, *p* < 0.01) dimensions, and the HADS D score being strong.

**Table 5:** Correlations between DERS. ERSQ. HADS and AEQoL scores

	1.	2.	3.	4.	5.	6.	7.	8.	9.	10.	11	12.	13.	14.	15.	16.	17.	18.	19.	20.	21.	22.	23.
1. ERSQ global score	-																						
2. ERSQ awareness	0.80**	-																					
3. ERSQ sensations	0.60**	0.43**	-																				
4. ERSQ clarity	0.79**	0.69**	0.44**	-																			
5. ERSQ understanding	0.84**	0.67**	0.47**	0.79**	-																		
6. ERSQ acceptance	0.70**	0.55**	0.29	0.42*	0.48**	-																	
7. ERSQ tolerance	0.80**	0.68**	0.42**	0.56**	0.58**	0.73**	-																
8. ERSQ self support	0.73**	0.59**	0.43**	0.65**	0.69**	0.38*	0.37*	-															
9. ERSQ confrontation	0.71**	0.46**	0.23	0.51**	0.49**	0.52**	0.61**	0.56**	-														
10. ERSQ modification	0.78**	0.53**	0.51**	0.53**	0.73**	0.40*	0.54**	0.68**	0.49**	-													
11. DERS global	−0.37*	−0.30	0.07	−0.15	−0.28	−0.33*	−0.45**	−0.25	−0.32	−0.43**	-												
12. DERS clarity	−0.38*	−0.30	−0.04	−0.29	−0.42**	−0.41*	−0.49**	−0.20	−0.21	−0.30	0.57**	-											
13. DERS goals	−0.11	−0.06	0.23	0.01	−0.10	−0.11	−0.18	−0.06	−0.13	−0.26	0.86**	0.34*	-										
14. DERS impulse	−0.45**	−0.46**	−0.02	−0.31	−0.46**	−0.34*	−0.45**	−0.32	−0.30	−0.36*	0.78**	0.45**	0.70**	-									
15. DERS strategies	−0.41*	−0.34*	−0.01	−0.16	−0.29	−0.31	−0.40*	−0.36*	−0.38*	−0.52**	0.88**	0.33*	0.78**	0.71**	-								
16. DERS non acceptance	−0.24	−0.26	0.02	−0.11	−0.16	−0.08	−0.30	−0.16	−0.08	−0.22	0.67**	0.34*	0.50**	0.53**	0.51**	-							
17. HADS anxiety	−0.27	−0.26	0.16	−0.20	−0.11	−0.35*	−0.45**	−0.15	−0.31	−0.06	0.48**	0.37*	0.27	0.34*	0.44**	0.53**	-						
18. HADS depression	−0.57**	−0.42**	−0.04	−0.42*	−0.50**	−0.41*	−0.41*	−0.56**	−0.58**	−0.51**	0.61**	0.48**	0.46**	0.46**	0.53**	0.31	0.42**	-					
19. AE-QoL total score	−0.26	−0.19	0.09	−0.14	−0.08	−0.45**	−0.36*	−0.12	−0.29	0.01	0.46**	0.27	0.41*	0.39*	0.35*	0.35*	0.64**	0.42*	-				
20. AE-QoL functioning	−0.20	−0.16	0.10	−0.11	0.04	−0.38*	−0.26	−0.12	−0.33*	0.11	0.26	0.12	0.19	0.28	0.21	0.14	0.49**	0.26	0.68**	-			
21. AE-QoL diet	−0.08	−0.04	0.19	0.01	0.07	−0.28	−0.19	−0.06	−0.14	0.09	0.37*	0.18	0.31	0.14	0.28	0.12	0.41*	0.28	0.66**	0.59**	-		
22. AE-QoL fatigue/mood	−0.45**	−0.35*	−0.09	−0.38*	−0.35*	−0.41*	−0.58**	−0.27	−0.36*	−0.21	0.54**	0.36*	0.46**	0.52**	0.42**	0.43**	0.48**	0.44**	0.80**	0.33*	0.54**	-	
23. AE-QoL fears/shame	−0.14	−0.13	0.05	−0.01	−0.02	−0.38*	−0.18	−0.02	−0.08	0.04	0.25	0.18	0.22	0.20	0.17	0.30	0.54**	0.24	0.80**	0.36*	0.24	0.51**	-

Note. * *p* < 0.05, ** *p* < 0.01. Abbreviations: HADS: Hospital Anxiety and Depression Scale; ERSQ: Emotion Regulation Skills Questionnaire; DERS: Difficulties in Emotion Regulation Scale; AEQoL: Angioedema Quality of Life Questionnaire

The links between emotion regulation difficulties (DERS-16) and anxious affect (HADS A) appeared to be positive for all dimensions except for the DERS goals dimension (Table 5). The link was strong for the DERS non-acceptance dimension (rho = 0.53, *p* < 0.01). All the DERS-16 dimensions, as well as the global score, were also positively associated with depressive affect (HADS D), with a strong correlation for the global score (rho = 0.61, *p* < 0.01).

### Emotion regulation and quality of life

Regarding quality of life (AE-QoL), negative associations mainly emerged with emotion regulation skills (ERSQ) for the fatigue/moods dimension of the AE-QoL, except for several ERSQ dimensions: sensations, self-support, confrontation, and modification. The link between the fatigue/moods dimension of the AE-QoL and ERSQ tolerance was strong (rho = −0.58, *p* < 0.01).

The global score of the AE-QoL was also positively correlated with all dimensions of the DERS-16, except for the clarity dimension. The fatigue/moods dimension of the AE-QoL was positively correlated with all dimensions of the DERS-16, as well as with the global score (rho = 0.54, *p* < 0.01) (Table 5).

## Perceived parenting style and emotion regulation

Perception of maternal care (PBI mother care) was positively and moderately correlated with ERSQ awareness (rho = 0.42, *p* < 0.05). No significant correlation appeared between the PBI mother care dimension and emotion regulation difficulties (DERS-16), Table 6. Similarly, the perception of maternal overprotection correlated only with the ERSQ modification score, both negatively and moderately (rho = −0.37, *p* < 0.05), but with no dimension of the DERS-16. The perception of paternal care (PBI father care) correlated only with the DERS strategies score (rho = −0.33, *p* < 0.05), negatively. The perception of paternal overprotection was moderately and negatively associated with ERSQ awareness (rho = −0.45, *p* < 0.01), ERSQ understanding (rho = −0.35, *p* < 0.05), and ERSQ tolerance (rho = −0.39, *p* < 0.05). However, it was positively correlated with the DERS strategies score (rho = 0.45, *p* < 0.01).

**Table 6:** Correlations between PBI, ERSQ and DERS scores

	1.	2.	3.	4.	5.	6.	7.	8.	9.	10.	11.	12.	13.	14.	15.	16.	17.	18.	19.	20.
1. PBI mother - care	–																			
2. PBI mother - overprotection	−0.56**	–																		
3. PBI father - care	0.33*	0.40*	–																	
4. PBI father - overprotection	−0.38*	−0.09	−0.25	–																
5. ERSQ global	0.28	−0.26	−0.14	−0.26	–															
6. ERSQ awareness	0.42*	−0.29	0.02	−0.45**	0.80**	–														
7. ERSQ sensations	0.13	0.02	−0.23	−0.04	0.60**	0.43**	–													
8. ERSQ clarity	−0.01	−0.09	−0.23	−0.24	0.79**	0.69**	0.44**	–												
9. ERSQ understanding	0.11	−0.26	−0.15	−0.35*	0.84**	0.67**	0.47**	0.79**	–											
10. ERSQ acceptance	0.05	0.05	−0.06	−0.03	0.70**	0.55**	0.29	0.42*	0.48**	–										
11. ERSQ tolerance	0.17	−0.14	−0.13	−0.39*	0.80**	0.68**	0.42**	0.56**	0.58**	0.73**	–									
12. ERSQ self support	0.305	−0.28	0.23	−0.21	0.73**	0.59**	0.43**	0.65**	0.69**	0.38*	0.37*	–								
13. ERSQ confrontation	0.27	−0.31	−0.01	−0.13	0.71**	0.46**	0.23	0.51**	0.49**	0.52**	0.61**	0.56**	–							
14. ERSQ modification	0.24	−0.37*	0.04	−0.22	0.78**	0.53**	0.51**	0.53**	0.73**	0.40*	0.54**	0.68**	0.49**	–						
15. DERS global	−0.05	0.16	−0.29	0.27	−0.37*	−0.30	0.07	−0,15	−0.28	−0.33*	−0.45**	−0.25	−0.32	−0.43**	–					
16. DERS clarity	0.14	−0.12	0.06	0.21	−0.38*	−0.30	−0.04	−0.29	−0.42**	−0.41*	−0.49**	−0.20	−0.21	−0.30	0.57**	–				
17. DERS goals	0.05	0.10	−0.29	0.27	−0.11	−0.06	0.23	0.01	−0.10	−0.11	−0.18	−0.06	−0.13	−0.26	0.86**	0.34*	–			
18. DERS impulse	−0.14	0.13	−0.24	0.31	−0.45**	−0.46**	−0.02	−0.31	−0.46**	−0.34*	−0.45**	−0.32	−0.30	−0.36*	0.78**	0.45**	0.69**	–		
19. DERS strategies	−0.11	0.20	−0.33*	0.22	−0.41*	−0.34*	−0.01	−0.16	−0.29	−0.31	−0.40*	−0.36*	−0.38*	−0.52**	0.88**	0.33*	0.78**	0.71**	–	
20. DERS non acceptance	−0.13	0.12	−0.21	0.45**	−0.24	−0.26	0.02	−0.11	−0.165	−0.08	−0.30	−0.16	−0.08	−0.22	0.67**	0.40*	0.50**	0.53**	0.51**	–

Note. * *p* < 0.05, ** *p* < 0.01. Abbreviations: ERSQ: Emotion Regulation Skills Questionnaire, DERS: Difficulties in Emotion Regulation Scale, PBI: Parental Bonding Instrument

### Impact of kallikrein inhibitors on alexithymia, anxiety–depression symptomatology, emotion regulation and quality of life

No significant difference was observed between patients receiving kallikrein inhibitors and those receiving other treatments. However, there was a trend toward significance in the ERSQ global score (*KS* = 1.31, *p* = 0.06), with patients on kallikrein inhibitors showing higher ranks (mean rank = 23.07) compared to those not receiving this treatment (mean rank = 16.23).

For all the other variables, differences did not reach significance, whether for alexithymia (TAS-20 overall score: *KS* = 1.01, *p* = 0.26; TAS-20 factor 1: *KS* = 1.02, *p* = 0.25; TAS-20 Factor 2: *KS* = 0.80, *p* = 0.54, TAS-20 Factor 3: *KS* = 0.88, *p* = 0.42), anxiety-depression symptomatology (HADS anxiety: *KS* = 0.58, *p* = 0.89; HADS depression: *KS* = 0.73, *p* = 0.65), emotion regulation skills subscales (ERSQ awareness: *KS* = 1.17, *p* = 0.13; ERSQ sensations: *KS* = 1.12, *p* = 0.16; ERSQ clarity: *KS* = 0.92, *p* = 0.36; ERSQ understanding: *KS* = 0.90, *p* = 0.39; ERSQ acceptance: *KS* = 0.64, *p* = 0.80; ERSQ tolerance: *KS* = 0.62, *p* = 0.83; ERSQ self-support: *KS* = 0.45, *p* = 0.99; ERSQ confrontation: *KS* = 0.72, *p* = 0.67; ERSQ modification: *KS* = 0.63, *p* = 0.82), or difficulties in emotion regulation (DERS global score: *KS* = 0.60, *p* = 0.86; DERS clarity: *KS* = 0.74, *p* = 0.64; DERS goals: *KS* = 0.81, *p* = 0.52; DERS impulse: *KS* = 0.51, *p* = 0.95; DERS strategies: *KS* = 0.68, *p* = 0.74; DERS non acceptance: *KS* = 0.44, *p* = 0.99).

## Discussion

The present study aimed to assess the presence of alexithymia and the links between alexithymia, emotion regulation skills, and emotion regulation difficulties in French adult patients with HAE. Our results showed that the frequency of definite alexithymia was low and that a 39.5% of patients had no alexithymia, based on the TAS-20 cut-off.

More than 30% of the patients in our study reported moderate to severe anxiety, and 7.7% reported moderate to severe depression. Overall, patients reported a fairly good quality of life and a moderate level of impairment. All emotional skills scores but the sensations dimension were negatively correlated with the overall alexithymia score. This score also appears positively correlated with emotions difficulties scores, except for the difficulties in goals, strategies, and non-acceptance subscales.

These results are consistent with those of Savarese et al. [45]. Interestingly, the proportion of patients with no alexithymia is lower in our study than in Savarese et al. study (39.5% vs 78%) The mean TAS-20 score was also higher in our population despite using the same tool and the same cut-off scores. These differences can be attributed to our sample comprising a higher proportion of male patients compared to that of Savarese et al. [45], as existing literature indicates that men tend to exhibit higher levels of alexithymia than women in non-clinical samples [55] as well as in cancer patients [56].

Regarding anxiety–depression symptomatology, our results align with results reported by Zarnowksi et al. [57], who also used the HADS to assess anxiety–depressive symptomatology, finding depression in 13.5% of their 37 patients with HAE and anxiety in 43.2%. Similarly, in an online study using the HADS, 38% of patients reported moderate to severe anxiety, while 17% reported moderate to severe depression [13]. Our results also confirm the study by Nicolas et al. [15], who show more anxiety than depression in French patients, with quite similar mean scores.

In terms of quality of life, our patient scores were also consistent with those reported by previous studies [13, 57]. Like Mendivil et al. [13], quality of life seemed to be the most affected with respect to the fear/shame and fatigue/mood dimensions. This finding may reflect the improvement in treatments in recent years [20]. The consistent findings that HAE patients show more anxious than depressive affect could reflect the persistence of fear of HAE attacks despite the improvement in treatments and medical care, as suggested before [15].

We also sought to determine whether adult patients with HAE exhibit difficulties in emotion regulation (e.g., difficulty identifying their emotional states). Regarding the presence of emotion regulation difficulties and poor emotion regulation skills, no norms or cut-offs on the ERSQ and DERS-16 scales exist. However, the ERSQ scores in our sample were very close to those described among American students by Grant et al. [50], in terms of both the overall and subscale scores. Like Grant et al. [50], our study confirms an association between alexithymia and emotion regulation skills (ERSQ) on the one hand and emotion regulation difficulties (DERS-16) on the other hand. Several studies in clinical sample have evidenced a prevalence of alexithymia and its relations with emotion regulation difficulties (DERS), anxiety and depression [58, 59]. Saure et al. [60] by example evidenced that patients with anorexia nervosa performed poorer in an emotion recognition task and had higher levels of alexithymia and emotion regulation difficulties in comparison with healthy controls, but only when anxiety and depression symptoms were not controlled for in the statistical analyses. Moreover most clinical studies focus in emotion dysregulation (e.g., DERS) rather that emotion regulation skills. In our sample—unlike the results of Grant et al. [50]—we did not find a significant correlation between TAS and ERSQ sensations. This suggests that, regardless of their level of alexithymia, HAE patients have good skills in terms of distinguishing bodily sensations linked to emotions. This may be related with the role of emotions as triggers in angioedema attacks [4–8, 61]. With regard to the DERS, we also note that difficulties in terms of emotion regulation strategies stand out from other types of difficulties.

To the extent that emotion regulation difficulties are also associated with depressive symptoms in our sample, this suggests that implementing interventions focused on emotion regulation and learning to use various strategies could help reduce depressive affect in patients [28, 62, 63]. The fact that anxious symptoms are not associated with emotion regulation difficulties suggests that other factors related to individual capacities—perhaps the frequency and severity of attacks—play a role in high anxiety.

It is also interesting and an innovative perspective to note that difficulties in accessing emotion regulation strategies are associated with paternal overprotection in our sample: the more participants reported a controlling paternal style, the more they reported difficulties in accessing these strategies. We also note an inverse pattern for the ERSQ: the more participants reported a controlling paternal style, the lower the overall emotion regulation skills score. These results therefore seem to support an impact of learning in terms of emotion regulation relative to the family context; HAE patients may have been discouraged from showing their emotions when they were children or adolescents, possibly to avoid triggering crises, and thus have fewer emotion regulation skills and more difficulty in mobilizing effective emotion regulation strategies as a result. But emotional suppression (i.e., behavioral inhibition of emotion suppression after the emotion is elicited [64]) has been by example showed to be associated with poorer psychological outcomes in cancer patients [65, 66]. On the contrary, coping through expressing emotions surrounding the disease with better outcomes [67]. This is consistent with the hypotheses proposed by Freda et al. [5] and Granero Molina et al. [68]. It is also possible that if one parent was affected by HAE and presented with anxiety–depressive symptoms, their parenting style was also affected, consequently impacting the patient’s emotion regulation [40]. It would be interesting to determine from a systemic perspective whether these results differ depending on who in the family is affected by HAE.

Finally, we aimed to determine whether patients with higher levels of alexithymia had fewer emotion regulation skills and experienced more specific emotion regulation difficulties compared to those with lower levels of alexithymia. Except for the sensations dimension, emotional skills scores were negatively correlated with the overall alexithymia score. Good emotion regulation skills can therefore be seen as a protective factor against alexithymia as well as depression and poor quality of life, given that the overall emotional skills score was positively associated with quality of life and negatively associated with depression. Regarding emotion regulation difficulties, the higher the overall difficulty score, the higher the alexithymia score; in this study, this was also the case for difficulties in terms of impulse (e.g., feeling out of control when upset) and clarity (e.g., having difficulty making sense of one’s feelings) in particular. However, difficulties in terms of goals (e.g., having difficulty thinking about anything else when upset), strategies (e.g., believing there is nothing to do to feel better when upset), and non-acceptance (e.g., being ashamed of feeling upset) did not appear to be correlated with the alexithymia score. In their study, Tombini et al. [69] used the DERS-36 with epileptic patients, also revealing a positive correlation between TAS-20 and global DERS, as well as with the subscales non-acceptance, goals, strategies, impulse, and awareness (e.g., “I am attentive to my emotions”). Similarly, Ayik et al. [70] showed that alexithymia in bipolar patients is associated with all dimensions of the DERS-36. In our study, the DERS subdimensions goals, strategies, and non-acceptance were not associated with alexithymia, which may be linked to our use of the shortened version of the DERS (i.e., the DERS-16), which does not assess the awareness dimension [71].

Our study has several limitations. First of all, the small number of patients does not allow the correlation of the findings with biological parameters (e.g., delayed diagnosis, preventive treatment, etc.). The sample size (39 patients) may seem modest, but it is important to bear in mind that HAE is a rare disease. There are currently about 1500 HAE patients in France, with 1000 followed in tertiary care centers. Our sample size is similar to other studies conducted in different countries with similar HAE prevalence [12, 13, 57, 72, 73]. As this study aims at exploring the psychological processes in patients with HAE, we did not include a control group. This choice may be questionable, but it seemed first irrelevant to use a control group made up of people who are not affected by the disease in their daily life, and second difficult to find a control group of patients with another rare disease from same age and same gender. This constitutes a notable limitation of our work. Moreover, as alexithymia global score correlated with depressive symptomatology, we cannot exclude a confounding effect of depression. Besides, the large number of bivariate correlations reported also increases the risk of Type I error, which constitute a statistical limitation. It would also have been interesting to investigate whether the scores differ between patients with high disease activity versus low disease activity, between patients being on prophylactic treatment (and the time they are on this treatment) versus on demand only, gender and age group. Comparing the scores of patients for whom stress is perceived as a trigger of their attacks with scores of patients who do not report this kind of trigger would also be a relevant issue in larger sample. It would be interesting to see whether the length of long-term prophylaxis influences anxiety, depression, and quality of life. Unfortunately, small sample size, one of the major challenges in studies conducted in rare diseases, did not allow us to run such analyses. Collecting data with multi-centers collaborations would help to overcome this limitation in future studies. Despite these limitations, our work provides exploratory data and preliminary insight into the relatively unexplored field of emotion regulation in HAE. Further studies performed on larger cohorts with a multi-center is warranted to consolidate our results.

Other limitations relate to the cross-sectional design and reliance on self-report measures. It should be kept in mind that the TAS-20 is a self-report measure and not a diagnostic tool, as a consequence results reflect tendencies in alexithymia, not clinical cases.

It would therefore be interesting to collect emotional data by using ecological momentary assessment in order to better appreciate the fluctuation in emotion regulation and psychological outcomes in the daily life of the patients, as well as triggers of the attacks [74].

Our sample also shows heterogeneity: some patients were diagnosed at 6 years, others at 55 years; some did not report any attack in the last six months, whereas others reported 20. However, this reflects the characteristics of the disease, which vary significantly from one patient to another. Some patients also benefited from treatment by kallikrein inhibitors, which considerably improves their quality of life. This might have affected the patients’ experiences, particularly regarding anxiety and depression [1, 75–77].

No significant differences were found between patients receiving antikallikrein inhibitors and those not receiving such treatment. This result is quite surprising, given that the literature reports improvements in quality of life for patients treated with kallikrein inhibitors [9, 22]. In our sample, there was substantial variability in the duration of antikallikrein treatment. This wide variation, combined with the small number of patients in each group, may account for the lack of observed differences between the two groups.

The results of this study highlight that patients with higher alexithymia scores have greater depressive symptomatology, fewer emotion regulation skills, more difficulties in emotion regulation, lower quality of life in terms of fatigue, and more overprotective paternal parenting style perceptions. These data allow us to better understand the importance to assess emotion regulation skills and difficulties. Indeed, given that these factors are linked to anxious–depressive symptomatology, implementing interventions focused on emotion regulation could effectively reduce anxiety and depression [63] and potentially lower levels of alexithymia [59, 78, 79]. An interesting approach would be to combine psychoeducation on emotions, as is already done in therapeutic education programs, with emotion skills training. Shared decision-making is a critical issue in HAE, particularly with the emergence of new treatments (e.g. [80, 81]). Alexithymia, by altering the perception and expression of emotions, may affect patients’ ability to identify and communicate their needs and preferences—a core component of shared decision-making. This could complicate patient-physician interactions, especially in individuals with high alexithymia tendencies. To our knowledge, the impact of alexithymia on shared decision-making in HAE has not yet been investigated. Addressing this research gap is important, as it carries significant clinical implications.

## Conclusions

This study examines the frequency of emotion regulation deficits in the form of alexithymia, as well as the association between alexithymia, emotion regulation skills, and emotion regulation difficulties, in order to identify whether any profiles emerge in French adult patients with HAE. Our findings show a low prevalence of definite alexithymia in HAE patients. Paternal parenting style that does not promote emotional experience (i.e., overprotection) reveals to be associated with more alexithymia. Higher level of alexithymia is associated with more difficulties and less skills in emotion regulation, higher depressive symptomatology and lower quality of life in terms of fatigue/mood dimension. These results suggest the potential role of interventions targeting emotion regulation features in HAE patients.

## Data Availability

The datasets generated and/or analysed during the current study are not publicly available to protect study participant privacy. They are available from the corresponding author on reasonable request.

## Abbreviations

AEQoL: Angioedema Quality of Life Questionnaire
AQC: Alexithymia Questionnaire for Children
C1INH: C1 inhibitor
DERS: Difficulties in Emotion Regulation Scale
ERQ: Emotion Regulation Questionnaire
ERSQ: Emotion Regulation Skills Questionnaire
GDPR: General Data Protection Regulation
HADS: Hospital Anxiety and Depression Scale
LEAS: Levels of Emotional Awareness Scale
M: Mean
N: Number of subjects
PBI: Parental Bonding Instrument
SD: Standard deviation
TAS 20: Toronto Alexithymia Scale-20
